# Presence of intracellular bacterial communities in uroepithelial cells, a potential reservoir in symptomatic and non-symptomatic people

**DOI:** 10.1186/s12879-024-09489-5

**Published:** 2024-06-17

**Authors:** Luciana Robino, Rafael Sauto, Cecilia Morales, Nicolás Navarro, María José González, Erlen Cruz, Florencia Neffa, Javier Zeballos, Paola Scavone

**Affiliations:** 1 Unidad Academica de Bacteriología y Virología, Instituto de Higiene, Facultad de Medicina, UdelaR, Av. Dr. Navarro 3051, Montevideo, Uruguay; 2 Laboratorio de Biofilms Microbianos, Departamento de Microbiología, Instituto de Investigaciones Biológicas Clemente Estable, Av. Italia 3318, Montevideo, Uruguay; 3 Servicio de Urología, Hospital Maciel, Montevideo, Administración de Servicios de Salud del Estado, 25 de mayo 174, Montevideo, Uruguay

**Keywords:** UTI, Intracellular bacterial communities, *E. coli*, *Stenotrophomonas maltophilia*, *Staphylococcus* spp., *Enterobacter cloacae*, Urinary microbiome

## Abstract

**Background:**

Urinary tract infection is one of the most common infections in humans, affecting women in more proportion. The bladder was considered sterile, but it has a urinary microbiome. Moreover, intracellular bacteria (IB) were observed in uroepithelial cells from children and women with urinary tract infections (UTIs). Here, we evaluated the presence of IB in urine from healthy people and patients with UTI symptoms.

**Methods:**

Midstream urine was self-collected from 141 donors, 77 females and 64 males; 72 belonged to the asymptomatic group and 69 were symptomatic. IB was characterized by a culture-dependent technique and visualized by confocal microscopy. Urine was also subjected to the classical uroculture and isolated bacteria were identified by MALDI-TOF.

**Results:**

One-hundred and fifteen uroculture were positive. A significant association was observed between the presence of symptoms and IB (*P* = 0.007). Moreover, a significant association between the presence of IB, symptoms and being female was observed (*P* = 0.03). From the cases with IB, *Escherichia coli* was the most frequent microorganism identified (34.7%), followed by *Stenotrophomonas maltophilia* (14.2%), *Staphylococcus* spp (14.2%), and *Enterococcus faecalis* (10.7%). Intracellular *E. coli* was associated with the symptomatic group (*P* = 0.02). Most of the intracellular *Staphylococcus* spp. were recovered from the asymptomatic group (*P* = 0.006).

**Conclusions:**

Intracellular bacteria are present in patients with UTI but also in asymptomatic people. Here, we report for the first time, the presence of *S. maltophilia, Staphylococcus* spp., and *Enterobacter cloacae* as intracellular bacteria in uroepithelial cells. These findings open new insights into the comprehension of urinary tract infections, urinary microbiome and future therapies. Uroculture as the gold standard could not be enough for an accurate diagnosis in recurrent or complicated cases.

## Introduction

Urinary tract infections (UTIs) are one of the most frequent infections in humans, affecting more than 50% of women worldwide at least once in their lives, and less frequently children and adult men [1].

Uropathogenic *Escherichia coli* (UPEC) is the main etiological agent, accounting for more than 80% of uncomplicated UTIs. During UTI, *E. coli* can adhere and invade the epithelial bladder cells, forming intracellular structures in the cytosol (termed intracellular bacterial communities IBCs) [2–4]. IBCs can protect bacteria from antibiotic action and serve as an intracellular reservoir for recurrent UTI [2–4].

Other bacteria genders and species, apart from *E. coli*, were identified in the cytoplasm of urinary epithelial cells from humans with UTIs. Some of these bacteria are: *K. pneumoniae* and *P. aeruginosa* [5–7].

Historically, urine in the urinary tract has been considered sterile (with the exception of the third distal of the urethra), documented by the absence of bacteria in standard urine culture methods. Contrary to this dogma, during the last ten years several studies have shown that urine is not sterile and a urinary microbiota is described, even in asymptomatic individuals [8, 9]. *Streptococcus spp*. and *Lactobacillus spp*. are the most frequent bacteria detected in healthy people [10]. Urine culture is the gold standard method for UTI diagnosis, however, in some patients with urinary tract symptoms bacteria are not detected by conventional urine culture. New methods like expanded urine culture and urine DNA sequence have detected the presence of bacteria in symptomatic patients with negative standard urine culture [9]. One explanation could be that the presence of intracellular bacteria, uncultured bacteria or less than 10e5 cfu/ml are not isolated/detected with conventional culture methods.

However, the presence of intracellular bacteria in the bladder epithelium as part of the human microbiota, in asymptomatic individuals, has never been studied. The aim of the present work was to evaluate and identify the presence of intracellular bacteria in desquamated cells in urine samples in a population with and without urinary symptoms.

## Materials and methods

### Study population and urine samples

The population was divided into two groups. One made up of voluntary individuals without urinary tract infection symptoms or urological pathologies (“Asymptomatic group”), and another population of patients with symptoms compatible with urinary tract infection (“Symptomatic group”) assisted at the polyclinic of urology of the Hospital Maciel or Centro Hospitalario Pereira Rossell, in Uruguay. Asymptomatic bacteriuria is defined as those individuals without symptoms, but with significant bacterial growth in urine culture. This group of individuals falls into the “asymptomatic group”. UTI is defined as the presence of symptoms and signs compatible with inflammation of the urinary tract, together with the presence of more than 100,000 CFU/mL in the urine culture. The inclusion of patients in the symptomatic group is independent of the urine culture result.

Both adult women and men were included. Antibiotic consumption during the last month was an exclusion criterion in both groups. For the “Asymptomatic group” one exclusion criterion was previous urinary tract infection records.

### Ethics, consent and permissions

All the procedures concerning human samples were evaluated and approved by the Ethical Committee in Human Research from IIBCE (CEI-IIBCE-004) and UdelaR (Exp Nº 070153-000360-19). The research was performed in accordance with the national regulations in Human Research. All the participants received an informed consent that was signed. After that clean midstream urine samples were obtained by each participant who previously received an explanation on how to take the sample correctly. The urine was kept at 4ºC without any additives, and all the procedures were done in the following 24 h. Once urine arrived at the laboratory a number was assigned in order to preserve the identity of the participants.

### Uroculture, bacteria cryopreservation and identification

Urocultures were performed on all urine samples. Ten microlitres of urine were cultured in blood agar and in Mc Conkey lactose agar, and incubated for 24 h at 37 °C. For the purpose of this study, ≥10^2^ UFC/ml was considered “positive” and bacteria were identified in order to evaluate correlation and similarities with the intracellular bacteria in those samples that intracellular bacteria were detected. Bacterial species identification was done using Matrix-Assisted Laser Desorption/Ionization (MALDI-TOF) (Bruker Daltonik microflex). All the isolates were cryopreserved in Brain Heart Infusion (BHI) Broth supplemented with 20% glycerol and kept at -80ºC.

### Evaluation of the presence of intracellular bacteria in desquamated cells from urine

Fourteen ml of urine were centrifuged (95 x *g*, 5 min, RT) and the supernatant was discharged. The pellet containing all the cells was resuspended in 1 ml of PBS, and from this 50 µl were spotted in blood agar as a first growing control. After that 100 µg/ml of Gentamicin was added and incubated for 2 h at 37ºC in order to kill all the extracellular bacteria. As a control of the effectiveness of the treatment 50 µl were spotted in Blood Agar and incubated for 24 h at 37ºC. The cells were centrifuged again (95 x *g*, 5 min, RT) and the supernatant was discharged. The pellet was resuspended in 10 ml of PBS, centrifuged (95 x *g*, 5 min, RT) and the pellet containing the cells was lysed with the addition of 0,1% of Triton X-100, incubated 15 min, 37ºC. 50 µl of the lysates were spotted in blood agar and incubated for 24 h, 37ºC. After this time, the blood agar plates were observed and all the colonies with different morphology in the last spot were subcultured for identification using MALDI-TOF (Bruker Daltonik microflex).

### IBC visualization by CLSM

Before IBC visualization by CLSM, all the urine samples were cytocentrifugated and stained with May Grunwald Giemsa in order to evaluate the presence of bladder-desquamated epithelial cells.

One ml of urine was cytocentrifuge (380 x *g*, 6 min) onto a glass slide and fixed with 4% PFA for 15 min. After that, fluorescence stains were performed as described before [3, 4]. Briefly, slides were stained with 1/50 goat anti-uroplakin III (UPIII, Santa Cruz Biotechnology) primary antibodies followed by incubation with the secondary antibody Alexa Fluor 568 donkey anti-goat immunoglobulin G for 15 min. After washing with phosphate-buffered saline (PBS), cell permeabilization was performed with 0,3% of Triton X-100 for 15 min. Then, the slides were incubated with 5.0 µg/ml 488-wheat germ agglutinin (WGA, Molecular Probes) and Hoescht 33342 (10 µg/ml) for 30 min. Once the incubation was over, the slides were washed with PBS and then mounted with 10 µl of citifluor.

3D Images stacks were obtained using a Zeiss LSM 800, with an oil 100X magnification (NA 1.4), 350/460, 488/520, 543/565 excitation/emission wavelength. Acquisition step was 0.3 μm on the z-axis and 1024 × 1024 pixels in xy-planes. The 3D image stack was reconstructed using ZEN Software (Zeiss) and Volocity (Perkin Elmer).

### Statistical analyses

The statistical analyses were performed using the SPSS 23 software (IBM SPSS Inc, Chicago, Illinois). The χ2 test or Fisher exact test were applied to nominal variables. P values < 0.05 were considered significant.

## Results

### Population

One hundred and forty-one people participated in the study. The gender distribution was 77 females (54.6%) and 64 males (45.4%). Sixtynine belonged to the symptomatic group (48,9%) and 72 were healthy asymptomatic. The population distribution is depicted in Table 1.

**Table 1:** Presence/Absence of urinary symptoms in the population of study (N = 141)

	Symptomatic (N = 69)	Asymptomatic (N = 72)
Female (N = 77)	45	32
Male (N = 64)	24	40
Total	69	72

### Standard uroculture

In 115 (81%) urocultures colony growth was observed; 56 (48,6%) in the symptomatic group and 59 (51%) in the asymptomatic group.

**Fig. 1:**
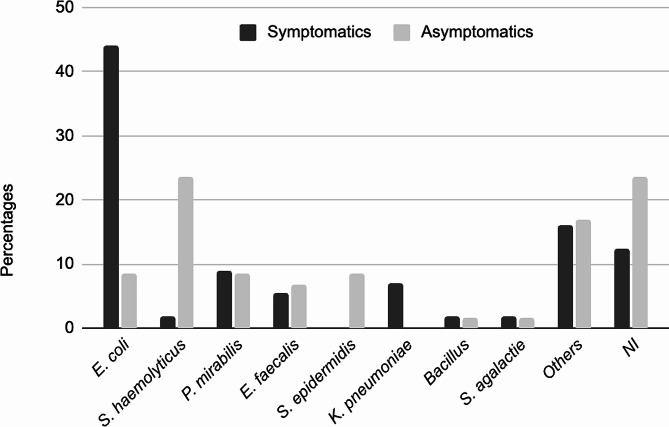
Percentages of the most frequently isolated microorganisms in urine by standard urine culture and distributed according to symptomatic and asymptomatic individuals. The category others refer to the case where a microorganism was isolated only one time. NI: No identity

The most frequent microorganisms isolated were *E. coli* 26%, *S. haemolyticus* 13%, *P. mirabilis* 9%, *E. faecalis* 6%, *S. epidermidis* and *K. pneumoniae* 4% each (Fig. 1). The frequency of the microorganisms identified in the uroculture was different depending on the population group. In symptomatics *E. coli* was the most frequent microorganism found in 44%, followed by *P. mirabilis* with 8,9% cases. In asymptomatics, *S. haemolyticus* was the most frequent microorganism found in urine with a frequency of 23,7%, followed by *P. mirabilis* and *S. epidermidis* at 8,5% each.

### Intracellular bacteria evaluation

The presence of intracellular bacteria was evaluated by a culture-dependent method developed in the present work. The method consisted of an extracellular bacteria-killing step and a cell lysis to release the intracellular bacteria. Each urine sample and each step of the procedure was cultured. As a control of the extracellular bacteria killing step (the second step) 50 µl were cultured in blood agar and no bacterial growth was observed in any case. The colonies in the third step were also subcultured and were identified by MALDI-TOF.

We observed the presence of intracellular bacteria in 72 of 141 urine samples (51.1%, Table 2), 42 were females and 30 were males. We observed a significant association between the presence of symptoms and the presence of intracellular bacteria (*P* = 0.007) as 43 patients had symptoms (65%) and 28 were asymptomatic (38%). The group that had symptoms and intracellular bacteria was constituted by 29 females and 14 males. We observed a significant association among the presence of intracellular bacteria, symptoms and females (*p* = 0.03). When we analyzed the group of asymptomatics with intracellular bacteria,13 were females and 16 were males. All these results are shown in Table 2.

**Table 2:** Intracellular bacteria and its associations with symptoms in the population of study. The χ2 statistical test was applied and *P* < 0.05 was considered significant

	Intracellular Bacteria			
	Presence (N = 72)	Absence(N = 69)	Total	*P*-value
Female with symptoms (N = 45)	29	16	45	**0.03**
Female asymptomatic (N = 32)	13	19	32	
Male with symptoms (N = 24)	14	10	24	0.12
Male asymptomatic (N = 40)	16	24	40	

All cytocentrifuged samples evidenced the presence of bladder-desquamated epithelial cells, some of them also showed bacteria, debris and immune cells. This reveals a great heterogeneity between the samples (data not shown).

**Fig. 2:**
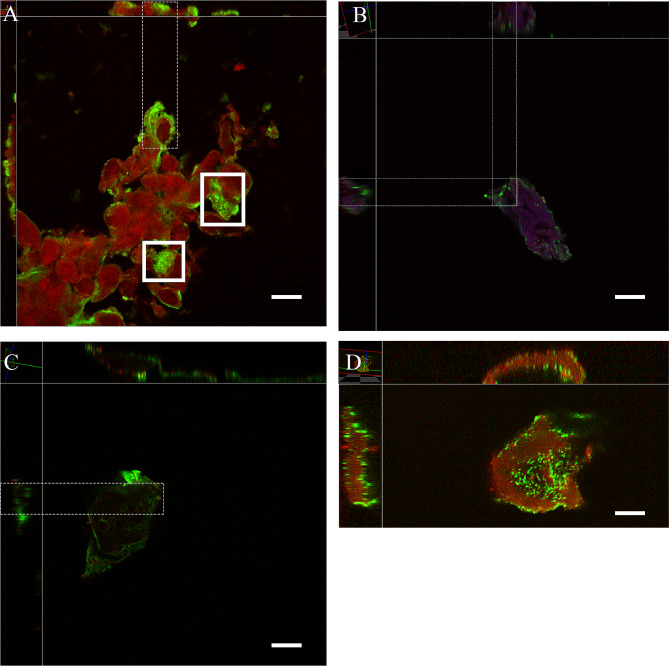
Confocal microscopy images of samples belonging to patients with intracellular bacteria (**A**) *K. pneumoniae*, (**B**) *S. haemolyticus*, (**C**) *S. maltophilia* and (**D**) *E. coli*. The bacteria were stained with 488-wheat germ agglutinin (green), goat anti-uroplakin III primary antibodies, Alexa Fluor 568 donkey anti-goat immunoglobulin G as secondary antibody (red) and Hoescht 33342 (blue). The scale bar represents 10 μm

The urine samples were also subjected to cytocentrifugation, immunofluorescence staining, image acquisition in confocal microscopy and observation of the presence of intracellular bacteria and its localization within the cells. Representative images are shown in Fig. 2. The fluorescence staining used consists of an antibody that recognizes uroplakin III (in red), bacteria (green) and DNA (blue). The image acquisition was done by another researcher independently of the intracellular identification in order to do a blind analysis. The images that had intracellular bacteria as depicted in Fig. 2, correspond to panel (A) *K. pneumoniae*, panel (B) *S. haemolyticus*, panel (C) *S. maltophilia* and panel (D) *E. coli.* In the case of *K. pneumoniae* it was possible to observe structures similar to intracellular bacterial communities and also different cellular morphologies corresponding to a great desquamation of the bladder epithelium or inflammation.

### Aetiological agents found intracellularly and in the standard uroculture

From the 72 cases with intracellular bacteria in 56, we could identify the aetiological agent (Table 3). In 16 samples we were unable to identify the microorganism, either bacteria did not grow after recovering from − 80ºC, or the MALDI-TOF result was unidentified. Also, in two patients we identified two aetiological agents intracellularly: *E. cloacae/E. coli* in one case and *E. faecalis/P. mirabilis* in the other case. Even though we only have two cases with two aetiological agents isolated from the same sample, these results show that more than one bacterial species can invade different eukaryotic cells in the bladder.

**Table 3:** Intracellular microorganisms identified by MALDI-TOF, result of the uroculture, presence of symptoms, gendre and other patient characteristics

Gendre	Symptomatic	Microorganism isolated in UCs	Intracellular microorganism	Patient characteristic
Male	No	No growth	*S. haemolyticus*	
Male	No	No growth	*K. pneumoniae*	
Female	No	No growth	*S. hominis*	
Male	No	No growth	*E. coli*	
Female	No	No growth	*E. coli*	
Male	No	*E. coli*	*E. coli*	
Female	No	*S. epidermidis*	*S. epidermidis*	
Male	No	*S. haemolyticus*	*S. haemolyticus*	
Female	No	*E. coli/ S. haemolyticus*	*S. haemolyticus*	
Female	No	*S.haemolyticus / S.lugdunensis*	*S. haemolyticus*	
Male	No	*E. faecalis / S. haemolyticus*	*S. haemolyticus*	
Male	No	*S. haemolyticus/ E. faecalis*	*E. faecalis*	
Female	No	*C. koseri/ P. penneri*	*P. penneri*	
Female	No	*S. haemolyticus*	*E. faecalis/* *P. mirabilis*	
Female	No	*Paenibacillus spp.*	*E. faecalis*	
Male	No	*E. faecalis/ M. morganii*	*P. mirabilis*	
Female	No	*E. faecalis*	*Bacillus iridensis*	
Female	No	*E. coli*	*Pseudogentamicibacter cumminsii*	
Female	No	*S. haemolyticus*	*E. coli*	
Male	No	*E. faecalis*	*Micrococcus luteus*	
Male	No	*S. epidermidis*	*S. maltophilia*	
Male	Yes	No growth	*E. faecalis*	uropathy
Male	Yes	No growth	*E. faecalis*	uropathy
Male	Yes	No growth	*S. maltophilia*	uropathy
Male	Yes	No growth	*S. maltophilia*	uropathy
Male	Yes	No growth	*S. maltophilia*	uropathy
Male	Yes	No growth	*S. maltophilia*	uropathy
Male	Yes	No growth	*Acinetobacter radioresistans*	uropathy
Male	Yes	No growth	*K. pneumoniae*	uropathy
Female	Yes	*S. maltophilia*	*S. maltophilia*	uropathy
Female	Yes	*Acinetobacter*	*S. maltophilia*	uropathy
Male	Yes	*P. aeruginosa*	*S. maltophilia*	uropathy
Female	Yes	*E. coli/ S. haemolyticus*	*E. coli*	
Female	Yes	*E. coli*	*K. variicola*	uropathy
Male	Yes	*E. faecalis*	*E. faecalis*	
Male	Yes	*S. capitis*	*E. coli*	
Female	Yes	*E. coli*	*E. coli*	
Male	Yes	*E. cloacae*	*P. mirabilis*	
Female	Yes	*E. coli*	*E. coli/E. cloacae*	
Female	Yes	*E. coli*	*C. kosseri*	
Female	Yes	*K. pneumoniae*	*E. coli*	
Female	Yes	*S. saprophyticus*	*S. saprophyticus*	
Female	Yes	*P.mirabilis*	*P.mirabilis*	
Female	Yes	*E. coli / E. faecalis*	*Bacillus iridensis*	
Female	Yes	*E. coli*	*E. cloacae*	
Female	Yes	*E. faecalis/ S. lugdunensis*	*E. coli*	
Female	Yes	*E. coli*	*E. coli*	
Female	Yes	*E. coli*	*E. coli*	
Female	Yes	*E. coli*	*E. coli*	
Female	Yes	*E. coli*	*E. coli*	
Female	Yes	*E. coli*	*E. coli*	
Male	Yes	*E. coli*	*E. coli*	
Female	Yes	*E. coli*	*E. coli*	
Female	Yes	*E. coli*	*E. coli*	
Female	Yes	*E. coli*	*E. coli*	
Female	Yes	*E. coli / E. faecalis*	*E. coli*	

Fifty-eight among 72 urine samples with intracellular bacteria had colonies in the standard uroculture. In 24/56 samples the same microorganism was identified intracellularly and in the uroculture. In 14/24 *E. coli* was found intracellularly and in the urine, in 6/24 were *Staphylococcus spp* present in both samples, in 2/24 *E. faecalis* and 1/24 *P. vulgaris* and *S. maltophilia*. In the case of the 14 samples with intracellular bacteria but without colonies in the standard uroculture, 8 were symptomatic (all males with uropathies) and 6 were asymptomatic (4 males and 2 females).

**Fig. 3:**
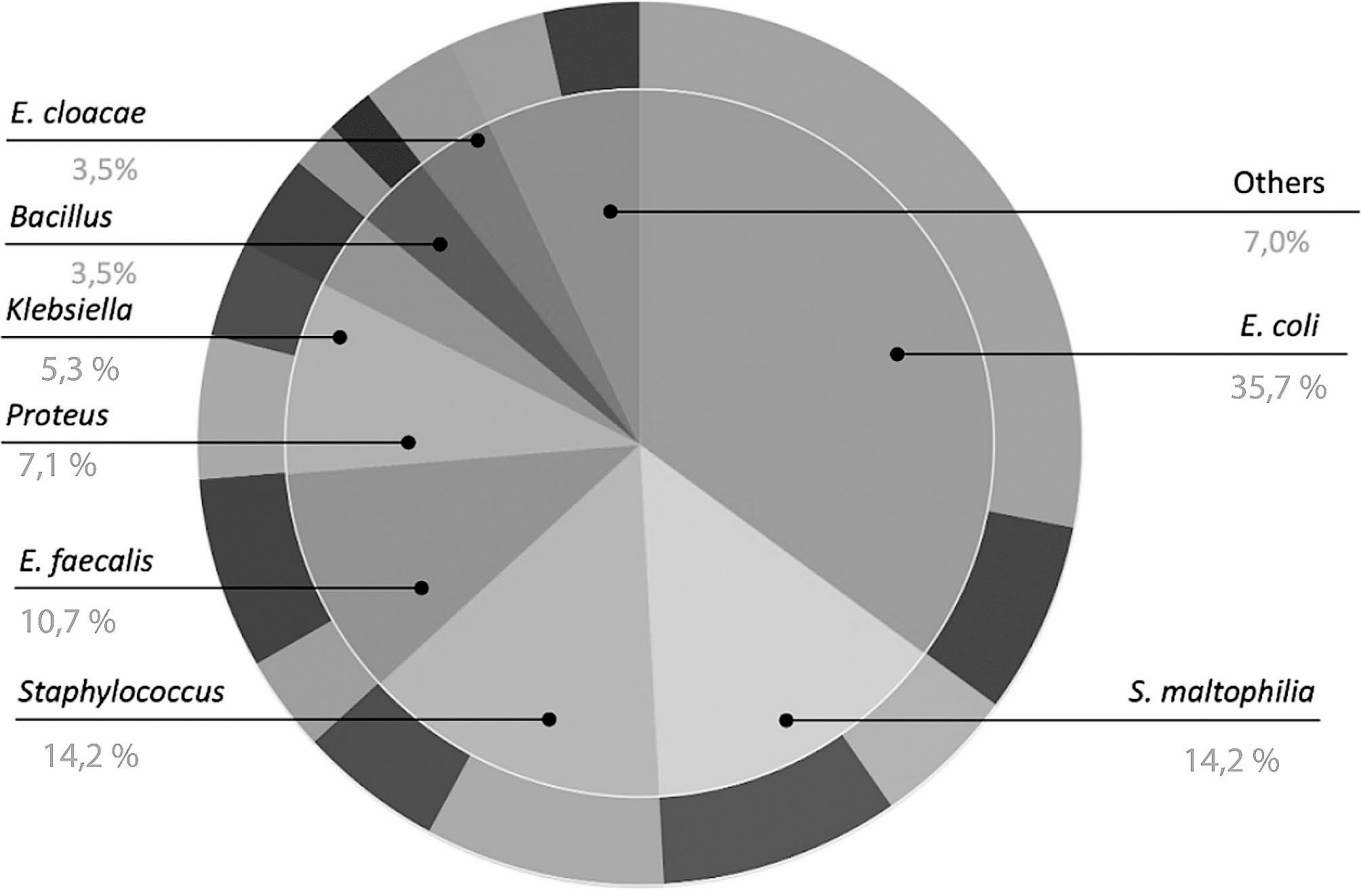
Distribution of the intracellular bacteria identified by MALDI-TOF. In the external part of the chart is represented the proportion of women and male that had the microorganism. Women are represented in clear colour and men in dark colour

The most frequent intracellular bacteria was *E. coli* in 20 samples (35.7%), followed by *Stenotrophomonas maltophilia* in 8 cases (14.2%), *Staphylococcus spp* in 8 samples (14.2%), *Enterococcus faecalis* in 6 (10.7%), *Proteus spp* in 4 (7.1%), *Klebsiella spp* in 3 (5.3%), *Bacillus* spp. in 2 (3.5%), and *Enterobacter cloacae* in 2 samples (3.5%) (Fig. 3). The other intracellular bacteria that appeared only in one case were *Acinetobacter radioresistens*, *Citrobacter koseri, Micrococcus luteus*, and *Pseudogentamicibacter cumminsiiare*.

In 16/20 samples where *E. coli* was detected intracellularly, the patients belonged to the symptomatic group (*P* = 0.02, Table 4). In the case of *S. maltophilia* 7 of 8 cases were also in the symptomatic group (*P* = 0.09), 5 were in symptomatic men (p 0.006), and the other 2 in symptomatic women. Only in one case, this microorganism was found in the standard uroculture. *Staphylococcus spp*. was also recovered from eukaryotic cells in 8 (13.6%) cases, 7 from the asymptomatic group (*P* = 0.006). The main species in this genera was *S. haemolyticus* (5), followed by *S. epidermidis, S. hominis and S. saprophyticus* (1 case each). *E. faecalis* was found intracellularly in 6 cases, 3 of them belong to the symptomatic group (*P* = 0.6). *Proteus* spp. was found in 4 (8.6%) cases and *Klebsiella* spp. in 3 (5.2%) cases, in both symptomatic and asymptomatic groups. The species recovered were *P. mirabilis* and *P. penneri* and *K. pneumoniae* and *variicola*.

**Table 4:** Distribution of the different intracellular microorganisms according to the presence of symptoms. The χ2 statistical test was applied and *P* < 0.05 was considered significant

Intracellular Bacteria (N: 72)	Symptomatic (N:43)	Asymptomatic (N:29)	*P*-value
*E. coli*	**16**	4	**0.02**
*S. maltophilia*	7	1	0.09
*Staphylococcus spp.*	**1**	**7**	**0.006**
*E. faecalis*	3	3	0.6
*Proteus spp.*	2	2	0.5
*Klebsiella spp.*	2	1	0.64

## Discussion

Urinary tract infection is one of the most common infections in humans [11]. The treatment of this infection is based on antimicrobial therapy, often empiric and based on symptom profiles without laboratory testing [12]. The use of antibiotics has led to bacterial resistance increase and the concomitant high recurrence of infection [13]. UTI affects mainly women and it is considered the most sex disparity infectious disease [14]. Several components as sex hormones, immune response, and microbiome composition would contribute to these differences [14].

Several works reported the presence of a urinary microbiota, now depicted as an urobiome consisting of bacteria, viruses and fungi [15]. The role of all these microorganisms in health and disease is not clear and the core of microorganisms in healthy people is not yet defined. Also, it was reported the presence of intracellular bacteria in the uroepithelium of women and children with urinary tract infections and other urological diseases [3, 4]. However, can intracellular bacteria be part of the urobiome in healthy people?

In this work, we report for the first time the presence of intracellular bacteria in the bladder of asymptomatic or healthy people as well as in patients with UTI symptoms. Although there is a greater significant association between the presence of intracellular bacteria and women with urinary symptoms we were able to detect intracellular bacteria in the other groups including non-symptomatic people.

Urine microbiome is not as rich as other microbiomes and contains 1 or 2 predominant bacteria genders. Depending on the predominant microbes there are different “urotypes” [16]. In healthy women, the most common urotypes described are those with predominance of *Lactobacillus*, *Gardnerella*, *Corynebacterium*, *Streptococcus* and *Staphylococcus* [17]. In our study, *Staphylococcus* spp. was the most frequent gender detected intracellularly in the asymptomatic group, even in women as in men. This gender was also detected extracellularly in most of these cases, supporting the concept of *Staphylococcus* as a member of a “healthy urotype” in women and men, living intra or extracellularly.

*Staphylococcus aureus* and *Staphylococcus* coagulase negative classically were considered extracellular pathogens. But in the last decade, it was demonstrated its capacity to internalize into both phagocytic and nonphagocytic cells, and now is accepted as a facultative intracellular pathogen [18, 19]. Its presence has been evidenced in cells of the respiratory epithelium, skin, and bone cells such as osteoblasts and osteoclasts. Up to our knowledge, it has not been described inside bladder cells. Some of the intracellular bacteria are small colony variants, with a quiescent metabolic state and a reduced immune response that increases their ability to persist in the host [19].

*E. coli* is the main etiological agent in UTI and also an intracellular bacteria producing IBC, which is related to the high recurrence of infection [2–5]. In this study, *E. coli* was the most frequent intracellular bacteria detected, and its presence was statistically associated with UTI, mostly in women.

Another microorganism detected intracellularly, mostly in symptomatic patients, was *S. maltophilia.* Intracellular *S. maltophilia* was associated with the group of symptomatic men. This bacteria is a gram-negative bacillus and is considered an emerging nosocomial agent, that causes mainly respiratory tract infections. In the last years, it has been reported as a UTI agent in particularly severe cases, as immunocompromised state, indwelling catheter, prolonged antibiotic treatment, and prolonged ICU stay. It can form biofilms that could be associated with an increase in antimicrobial resistance, along with all the reported resistance of *S. maltophilia*. The presence of intracellular *S. maltophilia* could serve as a reservoir for UTI as we detected the presence of this microorganism in symptomatic men that are not detected by common uroculture. In particular, the cases that had intracellularly *S. maltophilia* were patients with urological anomalies such as urethral constriction, who were assisted regularly in a health care centre and were exposed to different urological procedures.

Another intracellular pathogen that can invade bladder epithelial cells is *E. faecalis*. It was reported that the mechanisms that led to the internalization are different to the ones employed by *E. coli*. In particular, *E. faecalis* invasion is associated with hemolytic activity and its capability of forming biofilms [20]. Associated with the strong biofilm formation in *E. faecalis* is that it causes catheter-associated urinary infections (CAUTI) [21]. In long-term catheterization, it is reported the presence of polymicrobial infection and *E. faecalis* is thought to be one of the first species to colonize the urinary catheter. It is also reported that *P. mirabilis/E. faecalis* co-occurrence is one of the most prevalent and persistent in CAUTI [22]. Moreover, in the present work, we were able to observe one case of a co-invasion with *E. faecalis* and *P. mirabilis* confirming the findings that these two microorganisms co-colonize in humans, form strong biofilm association and live inside the eukaryotic cell. This finding should bring us an alarm regarding the treatment as this co-colonization could be a very difficult threat.

*E. cloacae* is not very frequent in UTI but it is part of the CESP (*Citrobacter, Enterobacter, Serratia* and *Providencia*) group responsible for serious health problems and associated with high antibiotic resistance rates [23]. One interesting aspect of this gender is the high rates of co-infections with other pathogens reported in liver and lung infections [23]. Here, we reported a co-occurrence of *E. cloacae* and *E. coli* intracellularly in a woman with UTI and *E. coli* infection reported by classical uro-culture. So far, this is the first report of the presence of *E. cloacae* intracellularly. The two cases that were reported here belong to two women with symptoms of UTI.

The present work brings new insights into UTI pathogenesis and contributes to the knowledge of urinary tract microbiota. The increased antimicrobial resistance will force us to improve the diagnosis methods and therapy. In these aspects, the results obtained here suggest that the uroculture -the gold standard- it is not enough to bring an accurate diagnosis in recurrent or complicated cases. The intracellular bacteria culture method developed for the identification of bacteria inside the cells could be included in clinical laboratories as it is cheap, easy to perform, and no specific knowledge or equipment are required. One limitation of this method is that only one culture media and incubation condition was used to recover intracellular bacteria. As it is not yet evaluated, a great improvement could be to use other media and anaerobic conditions as well as sequencing all the intracellular bacteria.

Moreover, new questions appear as healthy people have intracellular bacteria, most of them *Staphylococcus* spp. Are these intracellular bacteria protective against other uropathogenic microorganisms? How do healthy people with intracellular microorganisms control these reservoirs? Could new strategies that promote the control of these intracellular microorganisms prevent UTIs? Clinical-microbiological prospective studies and also urobiome research are needed to answer these questions.

## Data Availability

The datasets generated during and analyzed during the current study are available from the corresponding author on reasonable request.

## Abbreviations

CAUTI: Catheter associated urinary tract infection
CESP: Citrobacter, Enterobacter, Serratia and Providencia
CLSM: Confocal laser scanning microscopy
DNA: Deoxyribonucleic acid
IB: Intracellular bacteria
IBC: Intracellular bacterial communities
MALDI: Matrix-Assisted Laser Desorption/Ionization
PBS: Phosphate-buffered saline
PFA: Paraformaldehyde
RT: Room temperature
UPIII: Uroplakine III
UTI: Urinary tract infection
WGA: Wheat germ agglutinin
